# A computational workflow for the detection of candidate diagnostic biomarkers of Kawasaki disease using time-series gene expression data

**DOI:** 10.1016/j.csbj.2021.05.036

**Published:** 2021-05-24

**Authors:** Vasileios C. Pezoulas, Costas Papaloukas, Maëva Veyssiere, Andreas Goules, Athanasios G. Tzioufas, Vassili Soumelis, Dimitrios I. Fotiadis

**Affiliations:** a Unit of Medical Technology and Intelligent Information Systems, Department of Materials Science and Engineering, University of Ioannina, Ioannina GR45110, Greece; b Department of Biological Applications and Technology, University of Ioannina, Ioannina GR45100, Greece; c INSERM U976, Human Immunology, Physiopathology and Immunotherapy, Paris, France; d Department of Pathophysiology, School of Medicine, University of Athens, Athens GR15772, Greece; e Hôpital Saint Louis, Saint Louis Research Institute, Paris, France; f Department of Biomedical Research, FORTH (Foundation for Research & Technology)-IMBB (Institute of Molecular Biology and Biotechnology), Ioannina GR45110, Greece

**Keywords:** Systemic autoinflammatory diseases (SAIDs), Kawasaki disease (KD), Self-Organizing Maps (SOMs), Diagnostic biomarkers, Boosting ensembles

## Abstract

Unlike autoimmune diseases, there is no known constitutive and disease-defining biomarker for systemic autoinflammatory diseases (SAIDs). Kawasaki disease (KD) is one of the “undiagnosed” types of SAIDs whose pathogenic mechanism and gene mutation still remain unknown. To address this issue, we have developed a sequential computational workflow which clusters KD patients with similar gene expression profiles across the three different KD phases (Acute, Subacute and Convalescent) and utilizes the resulting clustermap to detect prominent genes that can be used as diagnostic biomarkers for KD. Self-Organizing Maps (SOMs) were employed to cluster patients with similar gene expressions across the three phases through inter-phase and intra-phase clustering. Then, false discovery rate (FDR)-based feature selection was applied to detect genes that significantly deviate across the per-phase clusters. Our results revealed five genes as candidate biomarkers for KD diagnosis, namely, the HLA-DQB1, HLA-DRA, ZBTB48, TNFRSF13C, and CASD1. To our knowledge, these five genes are reported for the first time in the literature. The impact of the discovered genes for KD diagnosis against the known ones was demonstrated by training boosting ensembles (AdaBoost and XGBoost) for KD classification on common platform and cross-platform datasets. The classifiers which were trained on the proposed genes from the common platform data yielded an average increase by 4.40% in accuracy, 5.52% in sensitivity, and 3.57% in specificity than the known genes in the Acute and Subacute phases, followed by a notable increase by 2.30% in accuracy, 2.20% in sensitivity, and 4.70% in specificity in the cross-platform analysis.

## Highlights

• Time-series microarray gene expression data were clustered using SOMs.
• Patients with Kawasaki disease were classified into four subgroups of similar genetic profiles.
• Five genes were identified across all three phases of the disease as diagnostic biomarkers.
• The proposed biomarkers performed better than the known on common and cross-platform datasets.

## Graphical abstract

**Figure:**
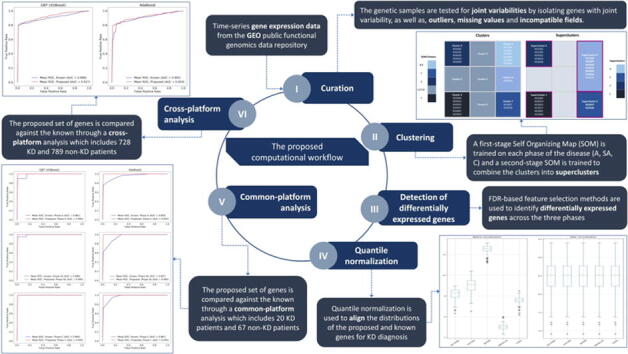

## 1 Introduction

Systemic autoinflammatory diseases (SAIDs) are a set of evolving groups of conditions sharing a core of phenotypical similarities [1], [2]. They encompass several rare disorders which have been characterized by extensive clinical and biological inflammation, with no specific age or gender distribution in the human population. Genetic mutations that may cause dysregulation of the innate immune system underlie the etiology of some SAIDs. Although they were proposed to constitute a continuum of disorders with potential overlap, SAIDs should not be confused with the autoimmune family of diseases, related to adaptive immune system dysfunction and response to self-antigen(s) [3]. Primary physical manifestations of SAIDs typically involve fever, rash, joint involvement, lymphadenopathy, and musculoskeletal symptoms. Due to the numerous symptoms observed in the different SAID-related conditions and their lack of specificity, diagnosis is challenging. Unlike autoimmune diseases whose autoantibodies are a tool for ascertaining the diagnosis, there is no known constitutive and disease-defining biomarker for SAIDs. Although inflammasome activation is thought to be a common pathophysiological pathway, the complex network of cytokine cascades together with multiple cell type activation makes difficult the use of these features as diagnostic or classification markers for SAIDs.

Kawasaki disease (KD) is a specific type of SAID which causes inflammation in the walls of medium-sized arteries throughout the body [4], [5], [6]. The underlying inflammation tends to affect the coronary arteries, which supply blood to the heart muscle and is the leading cause of pediatric acquired heart disease [7]. KD is also referred to in the literature as mucocutaneous lymph node syndrome (MLNS) because it also affects the glands, the skin tissues, and the mucous membranes inside the mouth, nose, and throat [8]. Signs of KD include high fever and peeling skin. KD mainly affects genetically susceptible infants and children. It has been characterized as an “undiagnosed” type of SAID for which no gene mutation has been identified so far and whose pathogenic mechanism remains unknown. Other examples of such SAID types include the neutrophilic dermatosis [9], and the recurrent pericarditis [10].

Apart from the conventional and widely used differential expression analysis which uses statistical approaches to identify statistically significant differences in the gene expression profiles, such as the Kruskal Wallis, machine learning (ML) has been deployed in the domain of autoimmune diseases for: (i) the molecular classification of patients with systemic sclerosis [11], (ii) the risk stratification of patients who have been diagnosed with systemic lupus erythematosus (SLE) [12], (iii) the prediction of Celiac disease [13], and (iv) the prediction of systemic lupus erythematosus using white-blood RNA-sequencing data [14]. Furthermore, ML has been used to shed light into the pathogenic mechanisms in rheumatic diseases [15], and for drug repurposing prediction in immune-mediated cutaneous diseases [16]. As far as SAIDs are concerned, there is a reported lack of significant scientific outcomes regarding the underlying pathogenic mechanisms. More specifically, ML has been deployed in two studies using Single Nucleotide Polymorphisms (SNPs) to predict intravenous immunoglobulin (IVIG) resistance in KD patients and discriminate those with higher risk of developing coronary artery abnormalities [17], as well as, to detect associations between post-IVIG IgG levels and clinical findings to understand the action of IVIG [18].

None of these studies, however, have reported outcomes which are related to the pathogenic mechanisms of KD. According to our knowledge, no studies have been reported so far regarding the analysis of Kawasaki disease by means of ML analysis on time-series based gene expression data. So far, most of the works in the field have focused only on the analysis of the associated SNPs [19], [20], [21]. In addition, the reported biomarkers for KD diagnosis have been experimentally determined in laboratory studies [22], [23] without the application of any data driven computational workflows. In KD, the diagnosis process is primarily clinical and relies on the collection of detailed patient’s history to fully understand the pattern of symptoms associated with the flair to categorize the patient’s condition. However, those criteria do not allow the definition of homogeneous groups of patients regarding the prognosis and response to therapy since even if a positive response to treatment is observed, it can be misleading as immunomodulating agents do not specifically target SAID mechanisms [24]. In addition, the lack of homogeneous groups of patients with KD regarding the underlying pathogenic mechanisms of the disease along with the discovery of data-driven biomarkers for KD development and diagnosis remain a clinical unmet need.

To address these needs, and mainly the need for KD diagnosis, we propose a computational pipeline which clusters KD patients with similar gene expression profiles across the three different KD phases, namely, the Acute (A), Subacute (SA) and Convalescent (C), and uses the resulting clustermap to detect prominent genes as biomarkers for KD diagnosis. To do so, we construct Self-Organizing Maps (SOMs) to group patients with similar gene expressions into homogeneous clusters across the three phases. Then, we apply FDR-based feature selection to detect genes that significantly deviate across the clusters on each phase. As a last step, we extract the final set of proposed genes as those that are present across all phases and compare their performance against known KD genes in the literature by training two ML algorithms for KD classification. According to the results, five prominent genes for KD diagnosis are proposed for the first time, namely the HLA-DQB1, HLA-DRA, ZBTB48, TNFRSF13C, and CASD1. These genes were used to develop a KD boosting classifier which yielded better performance against the one trained on the known KD genes in terms of increased accuracy, sensitivity, specificity, and AUC. To our knowledge, this is the first ML-based computational workflow using intra-phase and inter-phase clustering for KD genomic data analysis towards the discovery of biomarkers for KD diagnosis. Further examination of the proposed genes in terms of functional analysis, as well as, clinical validation may unveil new insights concerning the pathogenesis of KD and the underlying genetic mechanisms.

In the following sections, Section 2 describes the inter-phase and intra-phase patient clustering using SOMs, the extraction of the proposed set of KD genes, and the comparison process of the proposed KD genes against the known ones. Section 3 describes the results regarding the SOM prototypes, the proposed set of genes for KD diagnosis and the classification comparison between the known and the proposed KD genes. The obtained results and the derived findings are discussed in Section 4 along with a brief description of our future work in Section 5.

## 2 Materials and methods

### 2.1 Microarray data

Microarray data were collected from the Gene Expression Omnibus (GEO) public functional genomics data repository [25] for: (i) common platform analysis, where diagnostic biomarkers for KD are extracted from time-series gene expression data across three different KD phases followed by a validation of the extracted biomarkers against the known ones in the literature, and (ii) cross-platform analysis, where the proposed diagnostic biomarkers are further compared against the known KD genes through the integration of six more datasets. The clinical characteristics of all the patients are presented in detail in Supplementary Table 1.

**Table 1:** A summary of the datasets which participated in the common platform analysis.

**Platform**	**Dataset**	**Disorder**	**Values**	**Patient samples**
GPL6271	GSE9863 [26]	Kawasaki	Log2 median ratio	20 KD (at three phases)
GPL6271	GSE47683 [27]	Renal transplantation	Normalized log ratio	67 Non-KD (8 healthy subjects)

#### 2.1.1 Common platform data (with probes) for the detection of diagnostic biomarkers

The first dataset (GSE9863) includes 20 patients who have been diagnosed with Kawasaki across three different phases of the disease [26], namely, the A (Acute), SA (Subacute), and C (Convalescent), with a total number of 37,653 recorded genes per phase (size: 20x37653x3) which was used for the identification of the proposed diagnostic biomarkers for KD. The second dataset (GSE47683) consists of 59 patients who have been diagnosed with a different disease (renal-transplant patients) and 8 healthy subjects [27] (67 patients in total), with the same number of recorded genes (size: 67x37653). This dataset was utilized as the control group since it was the only dataset in GEO that uses the same experimental platform (i.e., GPL6271) like GSE9863 and thus the same gene probes can be used as input to the classifiers. Due to the significant lack of patient samples in GPL6271, the sample size was considered as adequate for the application of the proposed computational workflow.

#### 2.1.2 Cross-platform data for the validation of the proposed diagnostic biomarkers

In this case, the impact of the proposed set of diagnostic biomarkers for KD from the dataset GSE9863 (Table 1) was further evaluated against the known KD genes on six more datasets across two different platforms, namely the GLP570 and the GLP10558 (Table 2). It should be noted that these two platforms were selected as they both contain all the employed genes (proposed and known) of the current study. However, the corresponding probes are not identical with those of GPL6271, therefore the median expression value was extracted per gene, wherever many probes are referring to the same gene. According to Table 2, the six datasets consist of patients who have been diagnosed with Kawasaki along with other analogous diseases like systemic juvenile idiopathic arthritis (SJIA) or other inflammatory ones, as well as, autoimmune diseases like systemic lupus erythematosus (SLE), and other infectious diseases like Human Adenovirus (HAdV) and Group A streptococcus (GAS). These datasets were selected after a screening of GEO database using the following search terms - “KD” OR “Kawasaki” OR “SLE” OR “lupus erythematosus” OR “SJIA” OR “juvenile idiopathic arthritis”. Results were filtered to keep only whole blood datasets with at least 20 patients of the same disease. In the GSE68004 dataset, the 13 patients which were annotated as incomplete KD were excluded from the analysis. According to Table 1, the total number of patient samples in the cross-platform analysis was 1,347; 558 with KD and 789 as non-KD (154 healthy).

**Table 2:** A summary of the datasets which participated in the cross-platform analysis.

**Platform**	**Dataset**	**Disease**	**Values**	**Patient samples**
GPL570	GSE80060 [28]	SJIA	Linear scale RMA normalized relative expression values	206 Non-KD (22 healthy)
	GSE61635	SLE	RMA signal intensity in log2 scale	129 Non-KD (30 healthy)
GPL10558	GSE73461 [29]	KD, other inflammatory, bacterial/viral infections	Illumina calculated signal intensity	78 KD, 381 Non-KD (55 healthy)
	GSE63881 [30]	KD	Z-score normalization	171 KD (10 healthy)
	GSE68004 [31]	KD, HAdV, GAS	Average normalization	76 KD, 73 Non-KD (37 heathy)
	GSE73463 [29]	KD	Illumina calculated signal intensity	233 KD

### 2.2 The proposed computational workflow

**Fig. 1:**
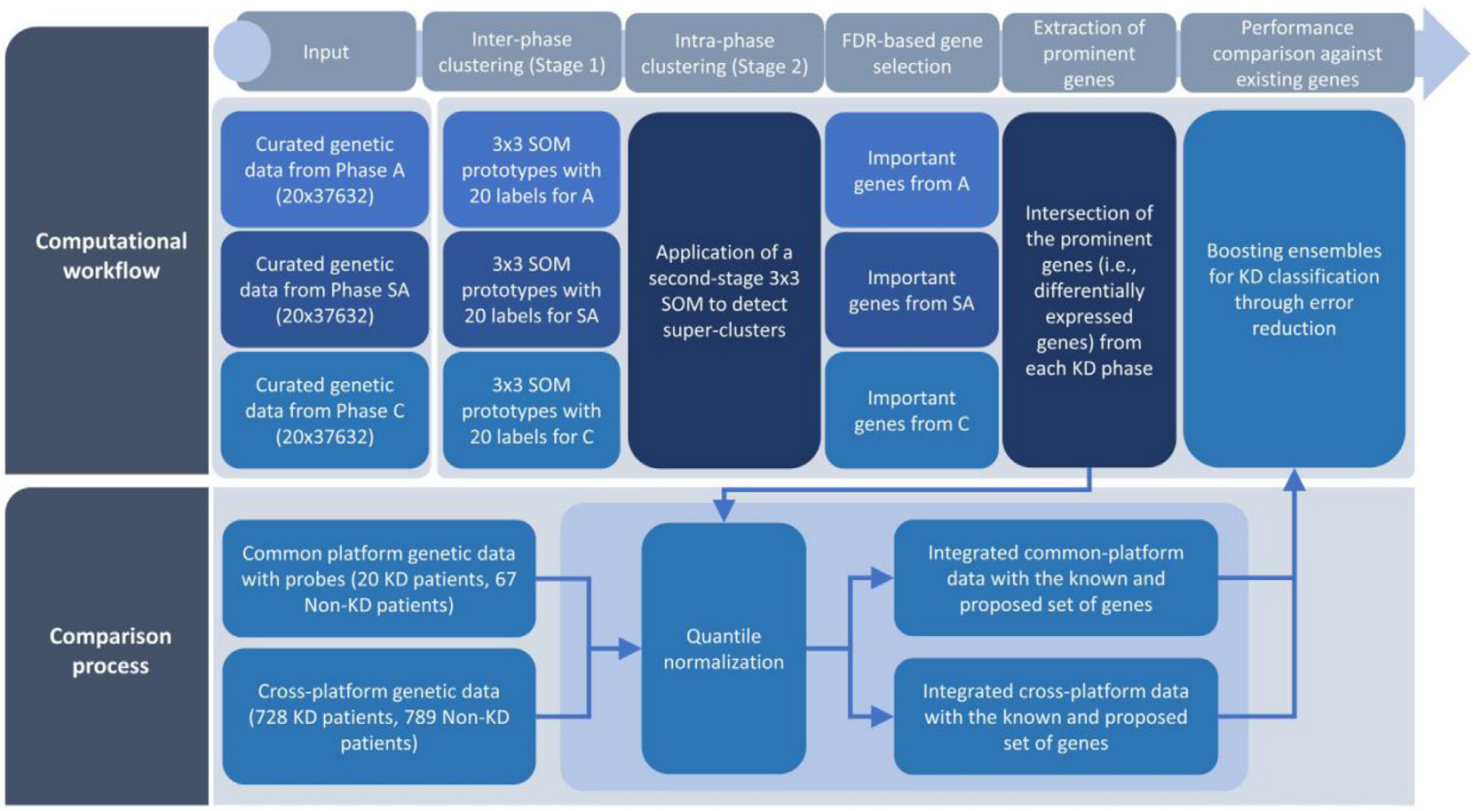
An illustration of the proposed computational workflow.

In this work, we focus on the development of a data-driven, computational workflow (Fig. 1) to provide new insights into the KD pathogenic mechanisms, through: (i) the inter-phase (or per-phase) clustering of KD patients with common genetic profiles across the Acute, Subacute, and Convalescent phases by constructing 3x3 Self-Organizing Maps (SOMs), (ii) the intra-phase clustering of KD patients by projecting the KD patients with similar per-phase clusters into a second stage SOM to detect super-clusters, (iii) the selection of important genes across each phase using the super-clustering labels of the second-stage SOM, as a target vector, by applying FDR-based feature selection, (iv) the extraction of prominent genes as those that are present across all three clinical phases, and (v) the performance comparison of the proposed KD genes against other known KD genes from the laboratory findings in the literature.

At the final stage (Fig. 1, Comparison process), the computational workflow is evaluated on common platform (on each KD phase) and cross-platform gene expression data from KD and Non-KD patients (Table 1, Table 2) using the proposed and the known KD genes, separately, for performance comparison. To do so, boosting ensembles are trained on each integrated dataset using error reduction. Performance evaluation measures are computed through a repeated stratified 10-fold cross-validation procedure to capture the performance of the ensembles without any biases during the training stage. The outcomes of the models are compared against their classification accuracy, sensitivity, specificity, and area under the ROC curve (AUC).

### 2.3 Genetic data curation and cross-platform *meta*-analysis

#### 2.3.1 Genetic data curation

An automated framework for data curation developed in a previous work [32] was adjusted to detect outliers and incompatible fields across time-series gene expression data structures. Multivariate methods, such as, the isolation forests and the local outlier factor were deployed to detect genetic samples that deviate from the standard distribution. The genetic samples were also tested for joint variabilities by calculating the covariance matrix and discarding genes with significantly high covariance. Any missing genetic samples were replaced with zero. Any incompatible fields and outliers were removed from the computational workflow to prior to the imputation process to avoid data contamination yielding high-quality genetic data.

#### 2.3.2 Meta-analysis

Due to the variation of the range of values across the microarray data which were obtained from the six datasets across the GPL570 and GPL10558 platforms (Table 2), as well as, from the two datasets in the GPL6271 (Table 1), a *meta*-analysis procedure was performed on each individual dataset based on the quantile normalization approach [33], [34]. Specifically, the average of each quantile across the proposed KD genes was used as the reference to transform (adjust) their distributions. The same process was applied on the known KD genes. Since one gene might have more than one probes, the median of the probes was extracted per gene, prior to the quantile normalization process.

### 2.4 Per-phase (inter-phase) patient clustering using Self-Organizing Maps (SOMs)

The first curated genetic dataset was sorted across the KD phases and concatenated onto the 3D space to formulate a 20x37653x3 data structure, where the 1st dimension corresponds to the patients, the 2nd dimension corresponds to the genetic samples and the 3rd dimension corresponds to the phases (i.e., A, SA, and C). Then, a SOM was trained on the samples from each phase to cluster the patients with similar genetic profiles. A Self-Organizing Map (SOM) [35], [36] is an unsupervised artificial neural network (ANN)-based method which reduces the dimensionality of the input data space into a lower dimension that represents the distributions of the data in the form of a map. The generated map, which is also known as Kohonen map, is a discretized version of the input space in the two-dimensional space, where each cell in the map represents a cluster. The clusters in the map are also referred to as prototypes.

SOMs generate low dimensional projections of the high-dimensional data using a training approach known as competitive learning. According to the competitive learning schema, the Euclidean distance is first computed between the input training sample and all the existing weight vectors of the neurons in the SOM. Then, the neuron with the smallest distance is extracted as the best matching unit (BMU). In fact, the BMU is the neuron whose weight vector lies closest to the input vector and is usually referred to as the winning neuron. The weights of the BMU and its nearest neurons are re-adjusted according to the following update function:

(1) $$w_{x}\left(i+1\right)=w_{x}\left(i\right)+U\left(x,y,i\right)\gamma \left(i\right)\left(x\left(q\right)-w_{x}\left(i\right)\right),$$

where $w_{x}$ is the weight vector of node x, i is the step (iteration number), q is the index of the input feature vector, u is the index of the BMU, x(q) is the input vector for the training sample with index i, γ(i) is the learning coefficient which is monotonically decreasing unproportionally of i, and $U\left(x,y,i\right)$ is the neighborhood function which calculates the distance between the neurons x and y, at step i. The neighborhood function is related with the grid-distance between the BMU and the input neuron x and shrinks over time since the weights converge to local estimates (local minima) at a step time T. The vectors that are close in the high-dimensional space also end up being mapped to SOM nodes that are close in the low-dimensional space. The overall process is repeated for each input vector and for multiple cycles until the grid shrinks, i.e., until the weight vectors converge to local minima (estimations). The algorithmic steps for SOM construction are summarized in Algorithm 1.

**Table:** 

**Algorithm 1.** A pseudocode for the construction of the inter-phase and intra-phase SOM.	
1	Generate a random weight vector $w_{x}$ for each node x in the 3x3 SOM.
2	Select a random feature vector as the input vector x'.
3	Calculate the Euclidean distance between the input vector and the weight vector.
4	Identify the node with the smallest Euclidean distance as the BMU.
5	Update the weight vector $w_{x\text{'}}$ according to (1).
6	Repeat Steps 2–5 until the maximum number of iterations is met or until the grid shrinks (i.e., the weights converge to local minima).

In the current work, the generated SOM consists of a rectangular 3x3 grid which is initialized on the input data yielding 9 clusters for the 20 patients. The topology of the grid was set to 3x3 since we noticed that some of the 9 clusters contained single or no samples at all and thus a larger grid size would be idle. Each sample in the grid corresponds to a patient with a multidimensional set of coordinates which is related to the genetic samples in the data. The development of the SOMs for inter-phase and intra-phase clustering took place in R 3.6.2 [37].

### 2.5 Combination of the per-phase clusters to create super-clusters (intra-phase clustering)

The resulting clustering labels from phases A, SA, and C, say $L_{A},L_{\mathrm{SA}},$ and $L_{C}$, respectively, were organized into a 20x3 data structure, say $L_{T}$, as follows:

(2) $$L_{T}=L_{A}\cup L_{\mathrm{SA}}\cup L_{C},$$

where the i-th row of $L_{T}$ corresponds to the i-th patient and the n-th column refers to the clustering label on the n-th phase, where n=1,2,3. An additional 3x3 SOM was applied on $L_{T}$ to group the patients with similar per-phase clusters yielding the final SOM. As a result, the per-phase clustermaps (first stage SOMs) were projected into a single clustermap (second stage SOM). Clusters with no samples were discarded from further analysis. The final clustermap was analyzed to detect super-clusters using hierarchical clustering with a dendrogram cut.

### 2.6 Extraction of prominent KD genes using FDR-based feature selection

The clustering labels from the final clustermap were used as a target vector to detect genes that contribute the most towards the precise discrimination of the KD patients among the superclusters. In each phase, the ANOVA (Analysis of Variance) F-value was computed between the feature vectors (genes) and the clustering labels (target vector) to examine the null hypothesis that there is no significant difference between the variance of the feature vectors and the target vector. Given an input vector z with N individual independent samples, the ANOVA F-score is defined as [38]:

(3) $$F=\frac{\sum_{i=1}^{N}n_{i}{\left(z_{i}-\overset{-}{z}\right)}^{2}/\left(N-1\right)}{\sum_{i=1}^{N}\left(n_{i}-1\right){s_{i}}^{2}/\left(n-N\right)}=\frac{\mathrm{MST}}{\mathrm{MSE}},$$

where $\overset{-}{z}$ is the mean of the input vector, N is the number of individual independent samples, and ${s_{i}}^{2}$is the variance of the i-th sample. The numerator in (3) is the treatment mean square (MST) which is equal to the variance between the N individual independent samples, whereas the denominator is the Mean Square Error (MSE) which is the variance within the samples. The F-test statistic assesses whether the N samples between the input vectors and the target vector are normally distributed with a common variance. If the population mean values between the input vectors and the target vector are the same, then the samples approximately follow an F-distribution with degree of freedom 1 equal to N-1 and degree of freedom 2 equal to n-N. The resulting p-values were adjusted using the Benjamini-Hochberg (BH) procedure with an alpha value set to 0.01 as an upper bound on the false discovery rate (FDR). The overall process was repeated for each phase, where genes with p-values larger than 0.01 were excluded from the pool of the proposed genes. The latter consists of the genes that appeared as significant across all phases. The implementation took place in Python 3.6.3.

### 2.7 Comparison of the proposed KD genes against the known ones in the literature

#### 2.7.1 Comparison process in the common platform analysis

The two datasets which were presented in Section 2.1.1 (Table 1) were integrated into a larger data structure with a size of 87x37657x3, where the proposed genes for KD diagnosis were evaluated against the known ones. For each phase, the initial data structure was split into two smaller subsets, namely A and B. Subset A includes only the proposed genes, with size mxk, where k is the number of the proposed genes and m is the total number of patients. On the other hand, the subset B includes only the known KD genes, with size mxl, where l is the number of the known genes. In both cases, m is equal to 87 patients. Boosting classifiers were trained on both subsets, separately, to develop a KD classification model. The two models were compared against each other in terms of their accuracy, sensitivity, specificity, and AUC.

#### 2.7.2 Comparison process in the cross-platform analysis

The six datasets (GSE80060, GSE61635, GSE73461, GSE63881, GSE68004, GSE73463) which were presented in Section 2.1.2 (Table 2) were integrated into two individual data structures for each type of genes (i.e., proposed and known), namely C and D. Subset C includes only the proposed genes, with size nxk, where k is the number of the proposed genes and m is the total number of patients, whereas subset D includes only the known KD genes, with size nxl, where l is the number of the known genes. In both cases, n is equal to 1,347 samples. The same boosting classifiers like in the common platform analysis were trained on both subsets, separately, to develop a cross-platform KD classification model where the same performance metrics were deployed to evaluate the classification performance of the models.

#### 2.7.3 Boosting through error reduction

Tree ensemble classifiers with boosting through error reduction, such as, the AdaBoost (Adaptive Boosting) [39] and the XGBoost (Extreme Gradient Boosting) [40] were deployed as robust supervised machine learning algorithms towards the development of the KD classification models. Boosting adopts a sequential strategy, where a set of weak learners is trained on the training subset and on each boosting round the next model learns from the errors that were made by the previous model. On each boosting round, the algorithm reweights the features according to the misclassification rate. Thus, features that misclassify the target receive a larger weight than the features with small misclassification rate. Then, the next model focuses on the features with the larger weights to improve the overall classification performance. The procedure is repeated until the number of boosting rounds is met.

The AdaBoost (Adaptive Boosting) classifier [39] is an ensemble classifier which combines a set of N-weak learners in a sequential error reduction fashion, where the final output of the classifier is a weighted sum of the weak classifiers. The final classifier can be expressed as:

(4) $$F_{N}\left(d\right)=\sum_{i=1}^{N}f_{i}\left(d\right),$$

where d is the input vector, $F_{N}\left(d\right)$ is the final classifier, $f_{i}\left(d\right)$ is a weak classifier, and N is the number of boosting rounds. The sequential version of (4) can be expressed as:

(5) $$F_{i}\left(d\right)=F_{i-1}\left(d\right)+a_{i}h_{i}\left(d\right)=F_{i-1}\left(d\right)+f_{i}\left(d\right),$$

where $F_{i}\left(d\right)$ is the ensemble at step i, $a_{i}$ is the weight that is given to the classifier at step i, and $h_{i}\left(d\right)$ is the outcome of the weak classifier at step i.

The Gradient Boosting algorithm [40] is also an ensemble classifier which combines a set of weak learners into a stronger classifier where on each boosting round the algorithm minimizes the gradient of a loss function to optimize the overall performance of the classifier. At step i the gradient boosting classifier seeks for a weak learner, say $f_{i}\left(d\right)$, so that:

(6) $$F_{i}\left(d\right)=F_{i-1}\left(d\right)+f_{i}\left(d\right).$$

Assuming that $\overset{\;}{y}$ is the predicted value at step i the goal is to minimize the cost function:

(7) $$F_{i}\left(d\right)=F_{i-1}\left(d\right)+{\mathrm{argmin}}_{f}\left(\sum_{j=1}^{n}L\left({\overset{\;}{y}}_{j},F_{i-1}\left(d_{j}\right)+f_{i}\left(d_{j}\right)\right)+r\right),$$

where ${\overset{\;}{y}}_{j}$ is the predicted value for the input sample $d_{j}$, L(.) is the error loss function, n is the number of samples, and r is a regularization term that is used to avoid overfitting. In the case of tree learners [40], the regularization term is defined as in:

(8) $$r=\gamma M+\frac{1}{2}\lambda \sum_{j=1}^{J}{w_{j}}^{2},$$

where γ,λ are scalars, M is the number of leaves in each tree learner, and w is the weight on the leaves. The implementation was performed in Python 3.6.3 [41] using the XGBoost [40].

## 3 Results

### 3.1 SOM prototypes

**Fig. 2:**
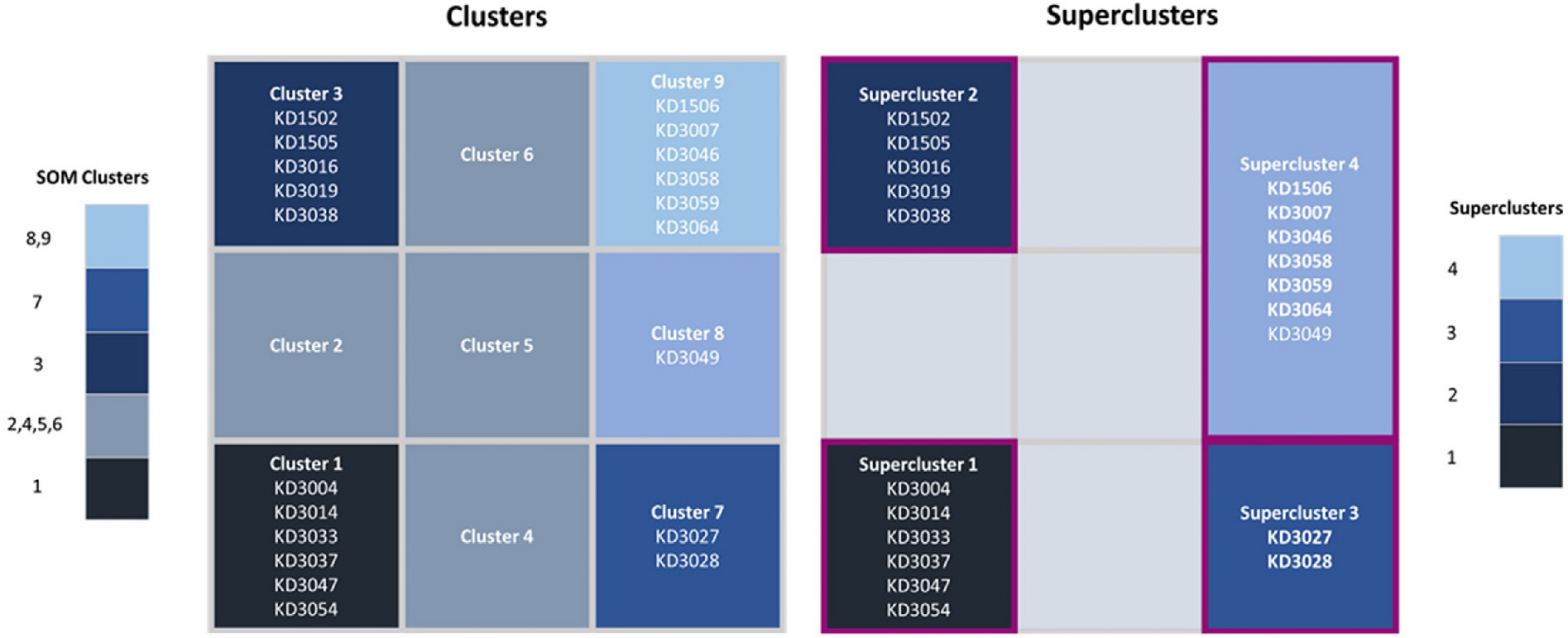
An illustration of the second stage SOM along with the detected super-clusters.

The rectangular grid of the second stage (intra-phase) SOM is depicted in the left-hand side of Fig. 2. The SOM consists of five clusters (prototypes), where, cluster 1 consists of six patients (KD3004, KD3014, KD3033, KD3037, KD3047, KD3054), cluster 3 consists of five patients (KD1502, KD1505, KD3016, KD3019, KD3038), cluster 7 consists of two patients (KD3027, KD3028), cluster 8 consists of one patient (KD3049), and cluster 9 consists of six patients (KD1506, KD3007, KD3046, KD3058, KD3059, KD3064). Note that the clusters 2, 4, 5, and 6 of the 3x3 SOM were empty since no samples were projected in those grid cells.

To merge prototypes with similar patterns, the five clusters were aggregated into super-clusters by applying hierarchical clustering on the Euclidean distances between them yielding the four prototypes (super-clusters) which are depicted in the right-hand side of Fig. 2. More specifically, the dendrogram which was generated by hierarchical clustering was partitioned into four super-clusters, where, super-cluster 1 consists of the patients in cluster 1, super-cluster 2 consists of the six patients in cluster 3, super-cluster 3 consists of the two patients in cluster 7 and super-cluster 4 is the union of clusters 8 and 9. The labels of the super-clusters were used subsequently to identify the proposed genes.

### 3.2 Proposed genes for KD diagnosis

The FDR-based feature selection schema was able to identify the following gene reference IDs as significant (p < 0.01, Benjamini-Hochberg adjusted) across all three phases: 15658, 15660, 22055, 26049, and 35359. The proposed genes for KD diagnosis are presented in Table 3. It should be noted that in order to map the gene IDs to the available gene probes and since the employed KD dataset does not provide any information on the utilized genes (ID, name or description) we performed a BLAST (Basic Local Alignment Search Tool) search on GenBank [42], to detect the most homolog sequence and subsequently the corresponding gene. The gene CASD1 achieved the highest score in phase A (F-score = 19.93), the gene TNFRSF13C in phase SA (F-score = 15.74), and the gene CASD1 again for phase C (F-score = 12.12).

**Table 3:** The proposed set of genes for KD diagnosis.

**ID_REF**	**Gene ID**	**ANOVA F-scores (with p < 0.01, BH-adjusted)**		
		**A**	**SA**	**C**
15,658	HLA-DQB1	9.79	10.05	10.51
15,660	HLA-DRA	11.12	9.95	9.03
22,055	ZBTB48	13.74	11.25	11
26,049	TNFRSF13C	12.22	15.74	11.59
35,359	CASD1	19.93	9.37	12.12

### 3.3 Classification comparison of the proposed KD genes against the known ones

#### 3.3.1 Known KD genes

The known KD genes from the literature are presented in Table 4 along with the corresponding ID_REF and a short description. Based on the work in [43] and the associated genes which are listed, we detected those that are also listed in [27] which, as already mentioned, uses the same experimental platform with the employed KD dataset but also provides the corresponding gene IDs. Probes with IDs 253, 29,567 belong to the TLR6 (Toll-like receptor 6) family which is related with pathogen recognition and activation of innate immunity. Probes with IDs 9368, 34,805 correspond to the COPB2 gene (COPI Coat Complex Subunit Beta 2) family which is part of the Golgi coatomer complex [44] that constitutes the coat of nonclathrin-coated vesicles and is essential for Golgi budding and vesicular trafficking. Probe ID 12,792 corresponds to the FCGR2A (Fc Fragment Of IgG Receptor IIa) which belongs to the family of immunoglobulin Fc receptor genes that exist on the surface of many immune response cells.

**Table 4:** Known genes for KD diagnosis.

**ID_REF**	**Gene ID**	**Description**
253	TLR6	Toll-like receptor 6 as plays a fundamental role in pathogen recognition and activation of innate immunity
29,567		
9368	COPB2	COPI Coat Complex Subunit Beta 2 constitutes the coat of nonclathrin-coated vesicles and is essential for Golgi budding and vesicular trafficking
34,805		
12,792	FCGR2A	Fc Fragment Of IgG Receptor IIa encodes a family member of immunoglobulin Fc receptor genes found on the surface of many immune response cells
26,186	CD40	The CD40 molecule belongs to the TNF-receptor superfamily and is a receptor on antigen-presenting cells of the immune system which is essential for mediating a broad variety of immune and inflammatory responses
33,880	BLK	BLK Proto-Oncogene is a protein which has a functional role in B-cell receptor signaling and B-cell development
37,136		
34,697	CASP3	Caspase 3 is a gene whose encoded protein is a cysteine-aspartic acid protease that plays a central role in the execution-phase of cell apoptosis

The probe with ID 26,786 is the CD40 molecule which is essential for mediating a broad variety of immune and inflammatory responses [44]. Probe IDs 33880, 37,136 belong to the BLK Proto-Oncogene family whose protein is involved in B-cell receptor signaling and development and finally the gene with ID 34,697 is the Caspase 3 (CASP3) which is highly involved in the execution-phase of cell apoptosis [44].

#### 3.3.2 KD classification outcomes

##### 3.3.2.1 Common platform analysis

**Fig. 3:**
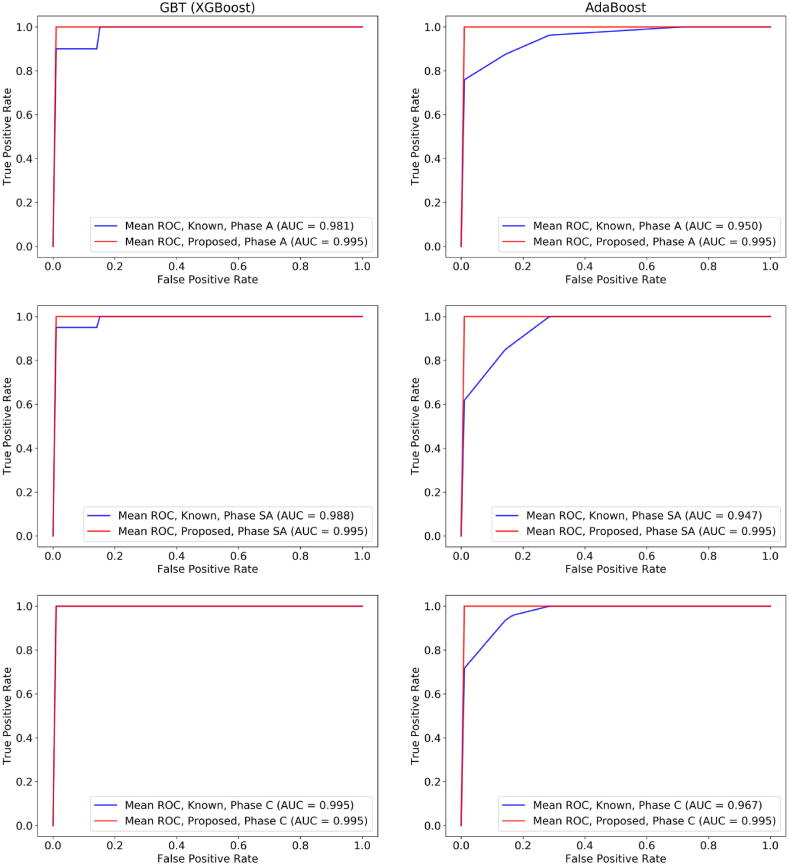
A comparison of the Receiver Operating Characteristic (ROC) curves (the true positive rate against the false positive rate) between the GBT (XGBoost) algorithm which was trained on the dataset with the proposed genes (red line) and the known KD genes (blue line), for phases A, SA, and C. (For interpretation of the references to colour in this figure legend, the reader is referred to the web version of this article.)

Each gene expression dataset from Table 1 was adjusted based on the quantile normalization process which was described in Section 2.2. No outliers or genes with joint variability were detected in the two datasets. The performance evaluation results of the XGBoost on both the proposed and the known genes are presented in Table 5. The procedure was repeated using the AdaBoost algorithm as a second boosting classifier to further compare the classification outcomes among the two cases (Table 5). A repeated stratified 10-fold cross validation procedure was applied for the performance evaluation of both boosting schemas, where four measures were averaged across the folds, namely, the accuracy, sensitivity, specificity, and AUC. Through the stratified strategy, the number of KD patients is the same across each fold. The corresponding ROC curves of the XGBoost and the AdaBoost are depicted in Fig. 3 for phases A, SA, and C and for each training case (case 1: on the dataset with the proposed genes and case 2: on the dataset with the known KD genes). In both boosting schemas, the proposed set of genes yielded a notable performance on the Acute and Subacute phases which is reflected by the high-performance evaluation results in Table 5.

**Table 5:** Performance evaluation results for the XGBoost and the AdaBoost across the three phases for both the known and the proposed set of genes.

**XGBoost**													
**Set of genes**	**Accuracy**			**Sensitivity**			**Specificity**			**AUC**			
	**A**	**SA**	**C**	**A**	**SA**	**C**	**A**	**SA**	**C**	**A**	**SA**		**C**
Known	0.956	0.989	0.989	0.918	0.975	0.975	0.986	1	1	0.981	0.988		0.995
Proposed	1	1	0.978	1	1	0.986	1	1	0.971	0.995	0.995		0.995
**AdaBoost**													
**Set of genes**	**Accuracy**			**Sensitivity**			**Specificity**			**AUC**			
	**A**	**SA**	**C**	**A**	**SA**	**C**	**A**	**SA**	**C**	**A**	**SA**	**C**	
Known	0.944	0.911	0.954	0.929	0.925	0.970	0.957	0.9	0.940	0.950	0.947		0.967
Proposed	1	0.976	0.989	1	0.968	0.993	1	0.986	0.986	0.995	0.995		0.995

Regarding the XGBoost algorithm (Table 5), the classification outcomes using the known set of genes yielded accuracy 0.956 for phase A, 0.989 for phase SA, and 0.989 for phase C, and the AUC scores were 0.981, 0.988, and 0.995, respectively (Fig. 3). On the other hand, the performance of the XGBoost on the proposed set of genes was higher in phases A and SA, yielding accuracy 1.0 for phase A and SA, and 0.978 for phase C, where the AUC scores were 0.995 across all phases (with a standard deviation ± 0.1). Although in phase C the sensitivity of the XGBoost on the proposed set of genes was 1.1% higher than the one on the known set of genes, the specificity was smaller thus yielding a slightly reduced performance.

As far as the AdaBoost algorithm is concerned, the increased performance of the proposed set of genes against the known ones is preserved, however, with an increased performance across all three phases. According to Table 5, the known set of genes yielded accuracy 0.944 for phase A, 0.911 for phase SA, and 0.954 for phase C, where the AUC scores were 0.950, 0.947, and 0.967, respectively. On the other hand, the performance of the AdaBoost algorithm on the proposed set of genes was higher in all phases, yielding accuracy 1.0 for phase A, 0.976 for phase SA, and 0.989 for phase C. The sensitivity values were 1, 0.968, 0.993 and the specificity values were 1, 0.986, and 0.986, respectively, yielding increased AUC scores across all phases.

In total, the classifiers yielded an average increase by 4.40% in the accuracy, 5.52% in sensitivity, and 3.57% in specificity compared with the known set of genes in phases A and SA. The contribution of the proposed set of genes appears to be significantly higher in phase A and SA, a fact which is also present in the AdaBoost schema. This implies that the high tendency of the proposed genes against the known genes is preserved in these two phases apart from the boosting schema. Regarding phase C, the high performance is maintained in the AdaBoost whereas in the GBT the reduced specificity results in a slightly smaller performance.

##### 3.3.2.2 Cross-platform analysis

Each gene expression dataset (GSE80060, GSE61635, GSE73461, GSE63881, GSE68004, GSE73463) from Table 2 was individually transformed (adjusted) using the quantile normalization process as it is described in Section 2.2. No outliers or genes with joint variability were detected. The median of the probes was extracted in the case of genes with more than one probes as described in Section 2.3.1. The transformed data were then integrated into two different data structures which included the proposed biomarkers and the known diagnostic biomarkers, respectively. The non-KD patients (including patients who have been diagnosed with SLE, SJIA or other inflammatory diseases, bacterial or viral infections, HAdV and GAS) were annotated with a value 0 whereas the KD patients were annotated with a value 1 to solve a binary classification problem using the XGBoost and the AdaBoost classifiers.

**Fig. 4:**
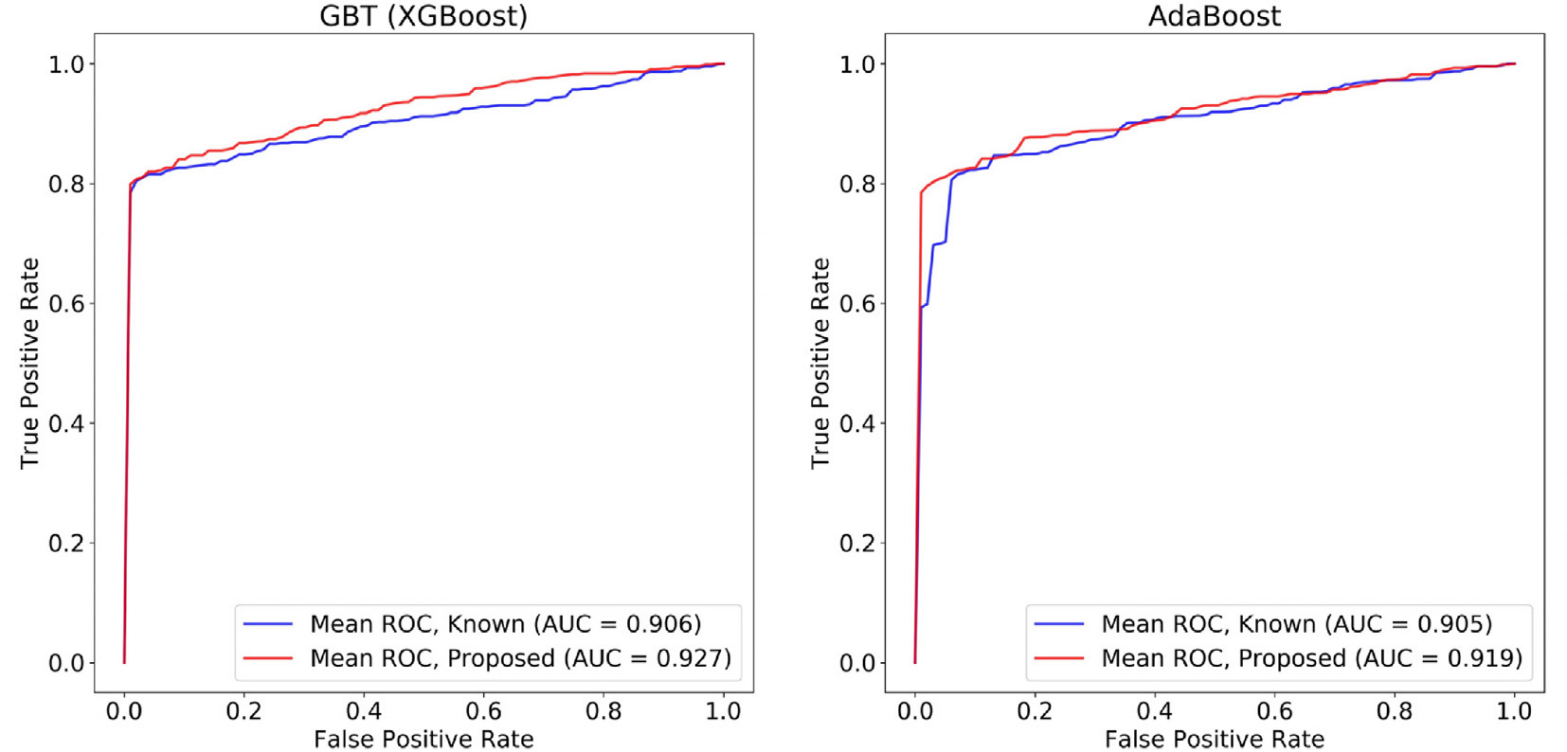
A comparison of the Receiver Operating Characteristic (ROC) curves (the true positive rate against the false positive rate) between the GBT (XGBoost) algorithm (on the left hand side) and the AdaBoost algorithm (on the right hand side) which were trained on the proposed genes (red line) and the known KD genes (blue line) across the cross-platform data. (For interpretation of the references to colour in this figure legend, the reader is referred to the web version of this article.)

Regarding the XGBoost algorithm (Fig. 4), the classification outcomes using the known set of genes yielded accuracy 0.847, sensitivity 0.845, specificity 0.894, and AUC 0.906, respectively. On the other hand, the performance of the XGBoost algorithm on the proposed set of genes was higher (Fig. 4), yielding accuracy 0.872, sensitivity 0.869, specificity 0.939, and AUC 0.927. As for the AdaBoost algorithm (Fig. 4), the increased performance of the proposed set of genes against the known ones is once more preserved, however, with a reduced performance than the XGBoost, like in the common platform analysis. The classification outcomes from the known set of genes yielded accuracy 0.848, sensitivity 0.846, specificity 0.892, and AUC 0.905. On the other hand, the performance of the AdaBoost algorithm on the proposed set of genes was higher (Fig. 4), yielding accuracy 0.868, sensitivity 0.865, specificity 0.94, and AUC 0.919. In total, both classifiers yielded an average increase by 2.30% in the accuracy, 2.20% in sensitivity, 4.70% in specificity, and in 1.70% in AUC.

## 4 Discussion

In this work, we present a data driven workflow for the analysis of time-series gene expression data in Kawasaki Disease (KD) towards the discovery of the underlying mechanisms for KD diagnosis through the formation of homogeneous clusters of KD patients with similar gene expression profiles across three phases, namely, the Acute (A), Subacute (SA) and Convalescent (C), and the detection of novel biomarkers for KD diagnosis. Self-Organizing Maps (SOMs) were constructed to group patients with similar gene expressions into concise inter-phase clusters. FDR-based feature selection was applied to detect genes that significantly deviate across the inter-phase clusters yielding the intra-phase clusters which in turn are grouped into super-clusters. The set of proposed genes is extracted as the set of the dominant genes across all phases. The performance of the proposed genes against the known KD genes, determined experimentally, was finally assessed by training two ML algorithms based on boosting ensembles for KD classification.

Our results reveal five prominent genes for KD diagnosis which are proposed for the first time, namely the HLA-DQB1, HLA-DRA, ZBTB48, TNFRSF13C, and CASD1. The KD classifiers which were trained on the proposed genes yielded better performance against those trained on the known ones, in terms of increased accuracy, sensitivity, specificity, and AUC. In the common platform analysis, the sample size in GPL6271 was considered as adequate for the application of the proposed computational workflow due to the significant lack of available KD patients, in terms of time-series expression profiling. To further test the discrimination performance of the proposed set of diagnostic biomarkers across other types of similar diseases, a cross-platform analysis was also conducted through the transformation and subsequent integration of six datasets from two different platforms (GPL570, GPL10558). The integrated dataset included 1,347 patient samples, where the non-KD group included patients with SJIA and SLE, which are characterized by certain clinical similarities with the KD patients. To our knowledge, this is the first data-driven workflow which constructs SOMs on the three clinical phases of KD based on time-series gene expression data towards the discovery of five candidate diagnostic biomarkers for KD with increased discrimination performance against other analogous diseases. The Self-Organizing Maps were constructed in a straightforward way to enable the clustering of the KD patients across the three clinical phases of KD, in a two-stage manner; the inter-phase and the intra-phase clustering. The two-stage clustering process yielded homogeneous and concise clusters of patients which were subsequently merged to identify four super-clusters. The derived super-clusters were able to categorize the available KD patients into four subgroups with similar genetic profiles across the whole duration of the disease and not on a single clinical phase to better comprehend the mechanisms of KD onset. The super-clusters were utilized, in a data-driven way, to extract the most prominent genes through FDR-based feature selection yielding statistically significant genes for KD diagnosis.

Both the boosting classifiers highlighted the impact of the proposed genes against the known KD genes, specifically in the Acute and Subacute phases, yielding an average increase by 4.40% in the accuracy, 5.52% in sensitivity, 3.57% in specificity, and 2.85% in the AUC. The performance of the AdaBoost on the proposed set of genes is significantly higher in all clinical phases of Kawasaki compared against the known set of genes. This increase, however, is not observed in the Convalescent phase for the GBT schema. These imply that the proposed set of genes can be used to shed light into the underlying pathogenic mechanisms and genetic basis of the KD onset with favorable precision in the first two phases of the disease. On the other hand, the known KD genes can be used to understand the evolvement of KD in the second clinical phase, where the patients already start to exhibit clinical manifestations and thus the pathophysiology is already observed. Regarding the cross-platform analysis, the boosting classifiers yielded an average increase by 2.30% in the accuracy, 2.20% in sensitivity, 4.70% specificity, and 1.70% in AUC, across the two boosting classifiers. This suggests that the proposed diagnostic biomarkers for KD present a notable discrimination performance of KD patients even in cases where the control group consists of patients that exhibit clinical similarities with KD. Finally, in both types of analyses, the gene expression data in the acute phase contribute most to KD prediction than those in the sub-acute and convalescent phases (Table 5) which is in line with the fact that early identification and timely IVIG (intravenous immunoglobulin) treatment is the best policy to treat KD.

The potential relation of the proposed genes with KD according to previous works reported in the literature is presented in Table 6. Specifically, for the HLA class II genes, like HLA-DQB1 and HLA-DRA, certain Single Nucleotide Polymorphisms have been associated with KD diagnosis in Genome Wide Association Studies (GWAS) reports [45]. Moreover, zinc finger proteins, like the ZBTB48, have been found to be down-regulated in KD patients [46], while increased TNFRSF13C gene expression has been associated with induced inflammation in RAW 264.7 cells [47]. Finally, several studies have indicated the role of CASD1 in the immune system [48], [49], [50]. These five genes are reported as biomarkers for KD diagnosis for the first time in the literature using data-driven analysis instead of the conventional laboratory analysis.

**Table 6:** Relation of the proposed set of genes with KD studies in the literature.

**ID_REF**	**GB_LIST**	**Gene ID**	**Description**
15,658	AI431505	HLA-DQB1	Association of the SNPs in HLA class II genes were documented as susceptibility genes of KD in GWAS reports [45]
15,660	AI434629	HLA-DRA	
22,055	AA810410	ZBTB48	Zinc finger protein 124 (circZNF124) has been found to be significantly down-regulated in untreated patients with Kawasaki disease [46]
26,049	AA864899	TNFRSF13C	TNFRSF13C is a target gene of miR-122 in RAW 264.7 cells’ inflammatory responses [47]
35,359	AI250844	CASD1	The role of CAS1 protein has been associated with the immune system in various works [48], [49], [50]

## 5 Future work

The present work mainly focuses on the development of a computational pipeline for the robust detection of candidate diagnostic biomarkers of KD based on time-series gene expression data. These markers can be used afterwards as targets in applications of qPCR (quantitative polymerase chain reaction)-based analysis for the biological validation of the KD prediction models [51]. Towards this direction, we plan to include such validation approaches on our future work, in order to provide a biological proof of concept regarding the proposed set of diagnostic biomarkers for KD. Moreover, the presented ML-based schema could be applied on other KD datasets derived from more recent experimental protocols, as well as, on more SAID-oriented genetic data to provide new insights on the underlying pathogenic mechanisms and biomarkers of SAIDs, such as, the Cryopyrin-Associated Autoinflammatory Syndromes (CAPS), the Hyperimmunoglobulinemia D syndrome (HIDS), and the Pharyngitis and cervical Adenitis (PFAPA), among others.

## CRediT authorship contribution statement

**Vasileios C. Pezoulas:** Conceptualization, Methodology, Software, Formal analysis, Writing - original draft, Data curation. **Costas Papaloukas:** Conceptualization, Writing - original draft, Writing - review & editing. **Maëva Veyssiere:** Investigation. **Andreas Goules:** Validation. **Athanasios G. Tzioufas:** Writing - review & editing. **Vassili Soumelis:** Validation, Writing - review & editing. **Dimitrios I. Fotiadis:** Writing - review & editing, Supervision.

## Declaration of Competing Interest

The authors declare that they have no known competing financial interests or personal relationships that could have appeared to influence the work reported in this paper.
