# Supplementary material for: A computational workflow for the detection of candidate diagnostic biomarkers of Kawasaki disease using time-series gene expression data
Source: Comput Struct Biotechnol J. 2021 May 24;19:3058–68. doi: 10.1016/j.csbj.2021.05.036 (PMC8178098; doi:10.1016/j.csbj.2021.05.036)
Supplement: Supplementary data 1 [file mmc1.pdf]

**Supplementary Table 1.** A summary of the age and gender distributions along with the study design and clinical characteristics of the patient subgroups across the datasets which were used in the common platform and the cross-platform analysis.

| **Platform/Dataset**[a] | **Study design and clinical characteristics** |
|---|---|
| GPL6271/GSE9873 [26] | • Whole blood samples were recruited from 20 individuals (14 males, 6 females).<br>• Median age: 3.5 (0.4-12.6) years.<br>• Immediately before treatment (acute), 11-24 days post-onset of fever (5-19 days post-IVIG treatment, subacute), and more than 24 days post-onset (convalescent). |
| GPL6271/GSE47683 [27] | • 67 Peripheral Blood Leukocytes (PLB) samples from 59 renal transplant patients (37 males, 22 females), 8 healthy subjects (8 females) with normal blood formulae and no infectious or other pathology for at least 6 months before the study.<br>• Average age of the patient group: 36.49 (10-87) years, Average age for the control group: 46 (30-66) years.<br>• The 59 renal transplant patients included TOL recipients (Tolerant transplant), CR (chronic rejection) patients, MIS patients (steroid monotherapy), STA patients (long-term stable test-group), and AR (acute rejection) patients. |
| GPL570/GSE80060 [28] | • 206 patient samples including 22 samples from healthy controls (11 males, 11 females) and 184 samples from patients with SJIA (88 males, 96 females). Out of the 184 SJIA patient samples, 33 samples were produced by placebo treated patients and 151 samples were produced by canakinumab treated patients.<br>• Average age of the patient and the control groups: 9 years.<br>• Patients were characterized by high levels of disease activity at baseline, 99.4% of 177 patients had a juvenile arthritis disease activity score (JADAS)-27 > 8.5, indicating high disease activity. |
| GPL570/GSE61635 | • Blood mRNA data from an SLE cohort with 129 patient samples 79 patients (73 females and 6 males) with some repeat visits for a total of 99 arrays and 30 healthy volunteers (one array per volunteer).<br>• The average/median age distribution of the patients was not available for all groups in the GEO repository along with the gender distribution for the healthy volunteers.<br>• Disease duration ranged from 0 to 453 months with a median of 37.5 months. SLE Disease Activity Index (SLEDAI) ranged from 0 to 31 with a median of 6. |
| GPL10558/GSE73461 [29] | • 459 patient samples were recruited, including: 78 patients with Kawasaki disease (43 males, 45 females) and 326 febrile control[b] samples (184 males, 142 females).The control group included: 55 healthy controls, 52 definite bacterial infection, 94 with definite viral infection, 84 with inflammatory diseases, 96 infections of uncertain bacterial or viral etiology.<br>• Average age of the patient group: 27 (16-45) months, Average age of the febrile control group: 37 (9-16) months.<br>• The median illness day at sample collection was 5 (4-6) for the KD patient samples and 6 (4-9) for the febrile controls, where illness day 1 is the first day of fever (in Kawasaki disease) or symptoms (in the case of the febrile controls). The main inclusion criteria included the presence of illness of sufficient severity to warrant blood tests in child aged < 17 years. |

| | |
|---|---|
| GPL10558/GSE63881 [30] | • Acute and convalescent whole blood transcriptional profiles of 171 KD patients, 10 healthy control children. The overall data in [30] included 146 KD acute samples (86 males, 60 females) and 131 convalescent samples (79 males, 52 females).<br>• Median age: 33 (4 - 182) months (for the KD patient samples), Median age of the convalescent samples: not available.<br>• The illness days in the 146 KD acute samples were 6 (2-10) and 51 (19-2,230) in the 131 KD convalescent samples, where illness day 1 refers to the first calendar day of fever. |
| GPL10558/GSE68004 [31] | • 113 patient samples were recruited (HAdV, cKD, FUO, GAS, GAS/SF and 37 healthy). According to [31], the overall data included 76 patient samples (44 males, 32 females) and 36 controls (15 males, 21 females).<br>• Average age of the patient group: 3.31 (1.4-5.9) years, Average age of the control group: 6.3 (1.9-12.7) years. |
| GPL10558/GSE73463 [29] | • 233 patient samples were recruited (146 acute and 87 subacute). According to [29], the overall dataset includes 72 KD patient samples (45 males, 27 females) and 130 febrile control[a] samples.<br>• Median age of the patient group: 34 (17-51) months, Median age of the febrile control group: 17 (5-47) months.<br>• The median illness day at sample collection was 5 (5-6) for the KD patient samples and 5 (3-7) for the febrile controls, where illness day 1 is the first day of fever (in Kawasaki disease) or symptoms (in the case of the controls). The main inclusion criteria included the presence of illness of sufficient severity to warrant blood tests, in child aged < 17 years. |
| [a] All eight employed datasets were obtained from GEO, therefore the required information is not available for all of them. The datasets were selected according to their gene probes, that is, they should include probes that refer to the 11 genes considered in our study.<br>[b] Healthy controls were not included in the demographic information. | |
